# l‐DNA Duplex Formation as a Bioorthogonal Information Channel in Nucleic Acid‐Based Surface Patterning

**DOI:** 10.1002/chem.202001871

**Published:** 2020-10-14

**Authors:** Erika Schaudy, Mark M. Somoza, Jory Lietard

**Affiliations:** ^1^ Institute of Inorganic Chemistry Faculty of Chemistry University of Vienna Althanstraße 14, UZA II 1090 Vienna Austria; ^2^ Chair of Food Chemistry and Molecular and Sensory Science Technical University of Munich Lise-Meitner-Straße 34 85354 Freising Germany; ^3^ Leibniz-Institute for Food Systems Biology Technical University of Munich Lise-Meitner-Straße 34 85354 Freising Germany

**Keywords:** l-DNA, microarrays, nucleic acids, photolithography, surface patterning

## Abstract

Photolithographic in situ synthesis of nucleic acids enables extremely high oligonucleotide sequence density as well as complex surface patterning and combined spatial and molecular information encoding. No longer limited to DNA synthesis, the technique allows for total control of both chemical and Cartesian space organization on surfaces, suggesting that hybridization patterns can be used to encode, display or encrypt informative signals on multiple chemically orthogonal levels. Nevertheless, cross‐hybridization reduces the available sequence space and limits information density. Here we introduce an additional, fully independent information channel in surface patterning with in situ l‐DNA synthesis. The bioorthogonality of mirror‐image DNA duplex formation prevents both cross‐hybridization on chimeric l‐/d‐DNA microarrays and also results in enzymatic orthogonality, such as nuclease‐proof DNA‐based signatures on the surface. We show how chimeric l‐/d‐DNA hybridization can be used to create informative surface patterns including QR codes, highly counterfeiting resistant authenticity watermarks, and concealed messages within high‐density d‐DNA microarrays.

## Graphical abstract

**Expanding the toolbox** for photolithographic in situ synthesis of microarrays to l‐DNA has opened an additional, independently accessible information channel. The mirror‐image DNA surface‐patterned information, including QR codes, authenticity watermarks and steganography, can thus be accessed in hybridization‐based assays using fluorescently labelled complementary probes without any interference with surface‐bound d‐DNA.

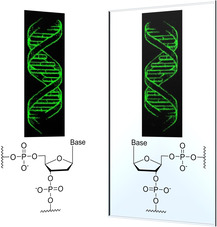

Oligonucleotide microarrays are versatile analytical tools where very large numbers of unique sequences are immobilized at precise locations on a planar surface to allow simultaneous access. Originally developed as platforms for gene expression analysis of cell populations,^[1]^ microarrays have recently found new applications in spatial transcriptomics,^[2]^ spatial organization of cell‐free genetic circuits,^[3]^ the generation of large oligonucleotide libraries for genomic applications,^[4]^ DNA circuitry,^[5]^ and others. In situ synthesized microarrays yield the highest oligonucleotide sequence density and, as such, are becoming an ideal source for the digital encoding of information in DNA.^[6]^ In addition, such array fabrication offers complete control over the spatial arrangement of sequences, suggesting that informative surface patterns may be created through simple hybridization‐based assays.^[7]^ Concomitant with the increasing throughput in DNA array synthesis and the decreasing costs of sequencing, there is greater access to DNA‐based information, which raises the potential question of privacy and traceability. It may thus soon become a necessity for data stored in nucleic acid format to provide an encryption layer or a traceability signature that is only available to the manufacturer and customer/operator. Such a key or signature could be produced in the form of binary matrices on the array itself and revealed via simple hybridization‐based assays, where 0=no hybridization and 1=duplex formation with a dye‐labelled complementary probe. Ideally, this key should be synthesized alongside the bulk information, but not interfere with it. We have recently expanded the method of maskless array synthesis (MAS)^[8]^ beyond native DNA, allowing for in situ synthesis of complex sequences containing 2′F‐ANA^[9]^ and RNA^[10]^ monomers, at high densities. However exotic, these nucleic acids are nonorthogonal to cross‐hybridization. While this can be mitigated by designing probes with low sequence similarity, temperature and salt concentrations can be tuned to force partial recognition. Our search for a truly orthogonal method that would not only prevent interaction with standard DNA but also provide an independently accessible information channel on the array led us towards mirror‐image DNA, the enantiomer of natural d‐DNA.

The d‐ and l‐DNA oligonucleotides of the same sequence have been shown to share common stability and solubility characteristics^[11]^ but differ in chirality, resulting in the formation of left‐handed B‐form duplexes for mirror‐image DNA compared to the right‐handed helical conformation in d‐DNA.^[12]^ Contradicting early reports regarding l‐DNA as a potential agent in antisense therapy,^[13]^ a key distinctive feature in l‐ and d‐forms is that hybridization exclusively occurs between oligonucleotide strands of equal chirality, eliminating the possibility of hybrid l‐/d‐DNA duplex formation.^[14]^ The absence of mirror‐image DNA in natural biological systems seems closely related to its increased stability against DNA‐degrading enzymes,^[15]^ which is an especially appealing feature of the use of l‐oligonucleotides in complex biological matrices.^[16]^ The bioorthogonality of mirror‐image oligonucleotides is indeed the basis for multiple applications, including the use of l‐DNA probes in PCR,^[17]^ the design of nanocarriers delivering d‐DNA aptamers to cells,^[18]^ recognition of small chiral molecules^[19]^ and the creation of heterochiral nucleic acid circuits.^[20]^ Whereas nuclease resistance is a central component of the bioorthogonal properties of l‐DNA, its inability to act as a substrate for natural l‐polymerases^[21]^ has hindered its use in molecular biology, despite recent efforts allowing for some key reactions to be performed using engineered d‐enzymes.^[22]^ A so far unexplored field for l‐DNA is in the storage of information. While data stored within DNA sequences can only be retrieved via sequencing, arrays of oligonucleotides allow for information to be communicated in the form of two‐dimensional binary grids upon hybridization with complementary labelled probes. The scale of MAS is determined by the number of digital micromirrors, and ranges from XGA (786 432 mirrors) to 4 K (8 847 360 mirrors), each mirror corresponding to a pixel where oligonucleotide synthesis can take place. Incorporating l‐DNA phosphoramidites in the process of photolithographic in situ synthesis introduces an additional information channel, which does not interfere with d‐DNA and which may be independently accessed. For these reasons, we intended to show how l‐DNA synthesis, along with d‐DNA synthesis performed in parallel (Figure 1 a), can serve to label surfaces with QR codes and watermarks for authentication, or to hide messages using steganography.

**Figure 1:**
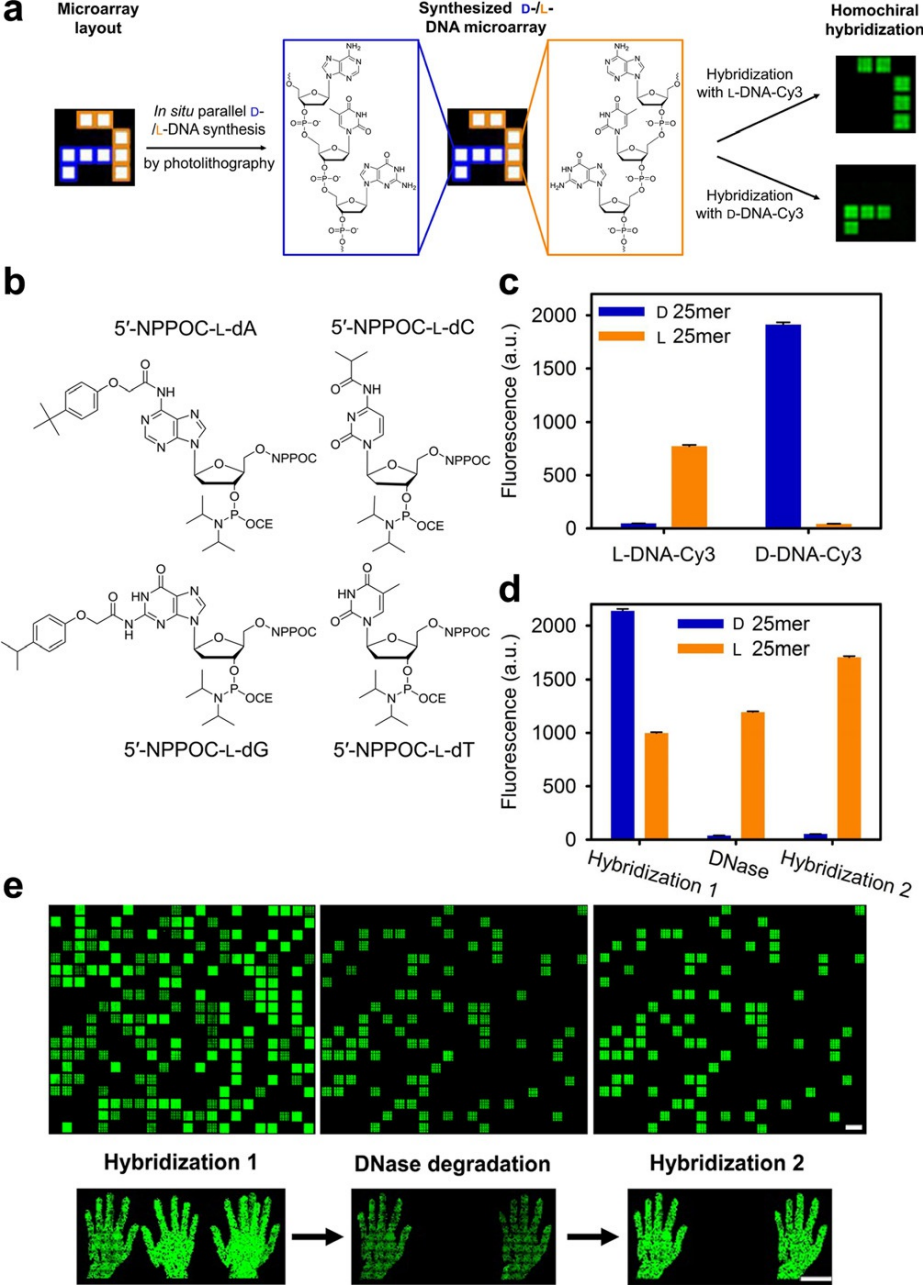
a) Schematic representation of a chimeric l‐/d‐DNA microarray with close‐up structural view of a trinucleotide section (3′‐GTA‐5′). b) Structures of l‐DNA phosphoramidites used in this study. c) Average signal intensities, in arbitrary units (a.u.), for hybridization of either l‐ or d‐DNA Cy3‐labelled probe to a l‐ (orange) or d‐DNA 25‐mer (blue) of the same sequence. d) Signal intensities for l‐ and d‐DNA after hybridization with a mix of Cy3‐labelled l‐ and d‐complement (Hybridization 1), following enzymatic degradation (DNase), and after rehybridization with the mix (Hybridization 2). e) Excerpt of scans of two arrays with either randomly distributed features (top) or l‐ and d‐pixels arranged in the shape of a left and a right hand (bottom), showing d‐DNA features with high signal intensities after initial hybridization (left), but disappearing after TURBO DNase treatment (center) and only l‐DNA features remaining after repetition of hybridization (right) (scale bars: 100 μm).

Initial experiments aimed to assess and evaluate coupling time,^[23]^ photolysis efficiency^[24]^ and stepwise coupling yield[^10a^, ^25^] of the 5′‐nitrophenylpropyloxycarbonyl (NPPOC) protected l‐DNA phosphoramidites (Figure 1 b), using Cy3‐labelled l‐ and d‐DNA complementary probes generated on separate microarrays (Figure S1). We found that a coupling time of 60 seconds resulted in a 30 % higher hybridization signal relative to a 15 seconds coupling time. Determining the light dose required for 95 % removal of the photolabile protecting group revealed a delayed photolysis of the NPPOC for l‐DNA monomers compared to their d‐DNA counterparts, requiring roughly 40 % higher light exposure to yield equal photodeprotection efficiency (Figure S3). Then, we measured the stepwise coupling efficiencies of each of the four l‐ and d‐monomers (5′‐NPPOC and 5′‐BzNPPOC‐protected, respectively). The results, shown in Table 1, indicate comparable coupling yields for corresponding l‐/d‐monomers.

**Table 1:** Comparison of the stepwise coupling efficiencies of l‐ and d‐DNA phosphoramidites (in %).

Phosphoramidite	dA	dC	dG	dT
5′‐NPPOC l‐DNA	99.9	99.9	98.3	99.9
5′‐BzNPPOC d‐DNA	99.9	99.9	97.0	99.9

Next, we wanted to examine the fundamental differences in the biophysical properties of l‐ and d‐DNA synthesized in situ on microarrays. To do so, we investigated specificity of hybridization as well as susceptibility towards an endonuclease by synthesizing the l‐ and d‐version of the same 25‐mer in parallel. First, two individual subarrays were hybridized with either an l‐ or d‐DNA complement. Figure 1 c shows hybridization taking place highly specifically to oligonucleotides of the corresponding chirality, with only background fluorescence levels for l‐/d‐chimeric hybrids, indicating that d‐ and l‐oligonucleotides of the same sequence do not interact with one another, which supports the restriction to homochiral duplex formation and which was previously reported on with mixed, spotted l‐ and d‐oligonucleotide arrays.^[15c]^ Since melting temperatures of homochiral l‐DNA duplexes have been shown not to differ significantly from those of natural DNA of the same sequence,[^11^, ^15c^, ^19^] the difference in signal intensity can be attributed to variations in purity and labelling efficiency of the two probes. We then studied the resistance of l‐DNA against nucleases (Figure 1 d). First, the l‐ and d‐sequences were hybridized to their complementary strands of similar chirality (Hybridization 1, Figure 1 e, left). The l‐ and d‐duplexes were then subjected to degradation using TURBO DNase, followed by rehybridization to a mixture of complementary enantiomers (Hybridization 2). Upon DNase treatment, all d‐DNA oligonucleotides were degraded, as signaled by the complete loss of hybridization fluorescence on d‐DNA features whereas l‐DNA duplexes remain bright (Figure 1 e, middle). The rehybridization step revealed clear l‐feature fluorescence only, showing that l‐DNA is not affected by the nuclease, whereas the fluorescence for d‐DNA features dropped by 98 %, to background level, as expected (Figure 1 e, right, and 1 d). These results validate the hybridization specificity and complete nucleolytic resistance of l‐DNA molecules when synthesized in situ on microarrays but, importantly, they show that d‐DNA synthesis can be performed alongside and become an “erasable” trace among “indelible” l‐oligonucleotides.

With no heterochiral hybridization taking place on the array and with mirror‐image DNA sequences withstanding nucleolytic treatment, we then applied l‐DNA in situ synthesis for the creation of informative surface patterns in three different contexts. In a first application, a QR code for a random 128 bit key made of l‐DNA was superimposed on a d‐DNA pattern. Restriction of duplex formation to homochiral complements resulted in the l‐DNA code remaining invisible upon hybridization solely with a d‐DNA probe. After addition of the l‐DNA probe however, the code appears (Figure 2 a) and resists endonucleolytic degradation (Figure 2 b).

**Figure 2:**
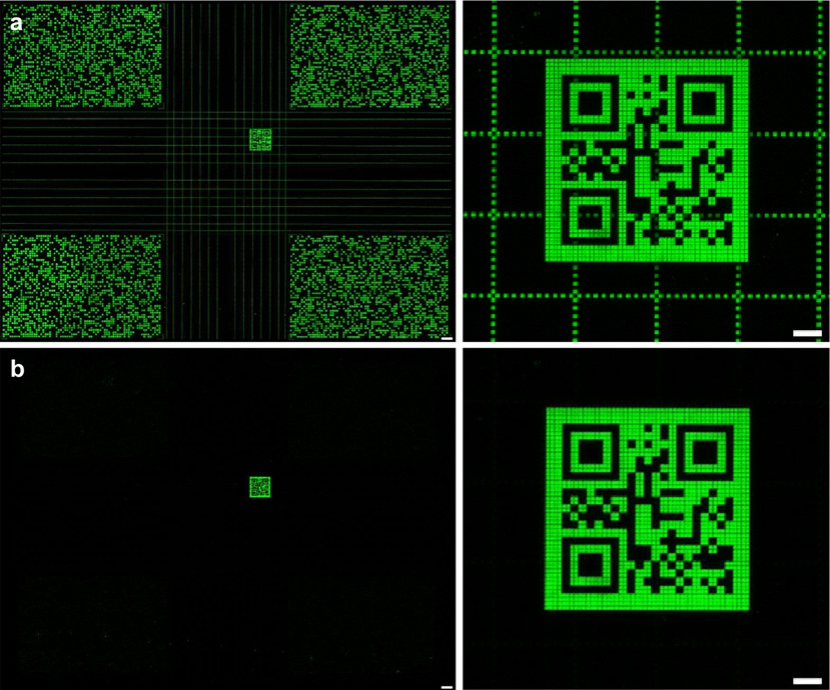
QR code introduced within a commonly used microarray layout. Hybridization with Cy3‐labelled d‐ and l‐DNA complement results in a d‐DNA grid crossing the code (a). Following endonuclease degradation, the l‐DNA code becomes clearly discernible (b) (scale bar full scan: 300 μm, close‐up: 100 μm).

Following our first attempts at producing informative, l‐DNA‐based patterns, we then generated d‐DNA microarrays supplemented with an l‐DNA authenticity watermark as a potential signature for microarrays originating from our laboratory. We followed an encryption scheme for oligonucleotide microarrays recently developed by Holden et al.^[26]^ The approach prevents a forger from deciphering a sequence using sequencing by hybridization (SBH), which is the only method allowing for sequence information to be retrieved while retaining the spatial ordering of oligonucleotide strands on the substrate. At the core of the approach, two individual oligonucleotide strands of high sequence similarity are combined within a single pixel, thus rendering SBH signals impossible to be assigned to only one of the strands. Inspired by this system, we produced the two strands/one feature combination by synthesizing two l‐DNA sequences in a row, spaced by a d‐DNA T_5_, thus creating a single 3′‐L_*x*_‐d‐L_*y*_‐5′ sequence instead of two individual strands. The d‐DNA spacer prevents sequence information retrieval through SBH via a discontinuity between the encoding strands. In a proof‐of‐concept, an array of 5×5 pixels at one corner of the microarray was used for l‐DNA synthesis to produce a distinct signal pattern upon hybridization with the correct key probe, whereas the remaining part of the synthesis area consisted of a d‐DNA 25‐mer (layout shown in Figure 3 a). The L_*x*_ and L_*y*_ sequences were generated as combinations of blocks of three specific 10‐mers (named A, B and C) according to the scheme and calculations discussed elsewhere^[26]^ (see Table S1). Here, creating an l‐DNA watermark allows for any other d‐DNA sequences to be addressed without the risk of interference with the signature. To create truly undecipherable 2D patterns, five different combinations of L_*x*_‐d‐L_*y*_ chimeras were designed (V1 to V5, setup according to Figure 3 c), resulting in the pattern shown in Figure 3 b upon hybridization with a single labelled l‐DNA probe (L_ABC‐complement_). An additional level of intensity is created through the introduction of background features (BG).

**Figure 3:**
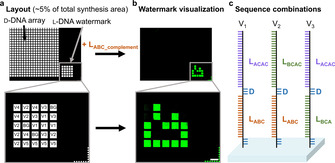
Arrangement and layout of a microarray with an authentication watermark. a) Layout and design of the array (≈5 % of entire synthesis area) and close‐up view of the pattern used for authentication. Each feature in the pattern contains 1 of 5 different combinations of two l‐DNA sequences (L_*x*_ and L_*y*_) separated by a short d‐DNA spacer. L_*x*_ and L_*y*_ are 30‐mers and 40‐mers, respectively, generated as combinations of 10‐mer blocks A, B and C. b) Scan after hybridization with the key L_ABC_complement_‐Cy3 (scale bar: 100 μm). c) Schematic view of some L_*x*_‐d‐L_*y*_ oligonucleotide combinations synthesized on the watermark (see Table S1 for sequences and combinations).

These complex watermarks would be particularly labor‐intensive to imitate because of three obstacles: sequence similarity between L_*x*_ and L_*y*_ preventing SBH, combinations of ABC blocks to which a given probe may or may not hybridize, and non‐hybridized features being equivalent to background. Furthermore, l‐DNA sequence identity cannot be recovered by cleaving and isolating the l‐oligonucleotides, even after sacrificing spatial information, since current high‐throughput sequencing methods for the analysis of large oligonucleotide libraries rely on the use of l‐enzymes and are therefore not applicable to l‐DNA base‐calling.

Finally, we applied l‐DNA in steganography as a way to conceal a message within a photographic reproduction composed in d‐DNA with a resolution of 1024×768 pixels. The message is encoded in decimal form on the *x* coordinates of l‐DNA pixels. The premise of the approach is based on the assumption that a few additional features lighting up would seem inconspicuous to the naked eye, yet would be identifiable by standard data extraction. The pattern visible after initial hybridization with a complementary d‐DNA probe indeed does not suspiciously differ from the version after hybridization with a mix of d‐ and l‐DNA probe (Figure 4 c d). Aligning scans with the underlying microarray design followed by data analysis allows for the coordinates of the pixels displaying unusual florescence to be recognized. The hidden message (Figure 4 e and Table S2) can then be retrieved using an ASCII table.

**Figure 4:**
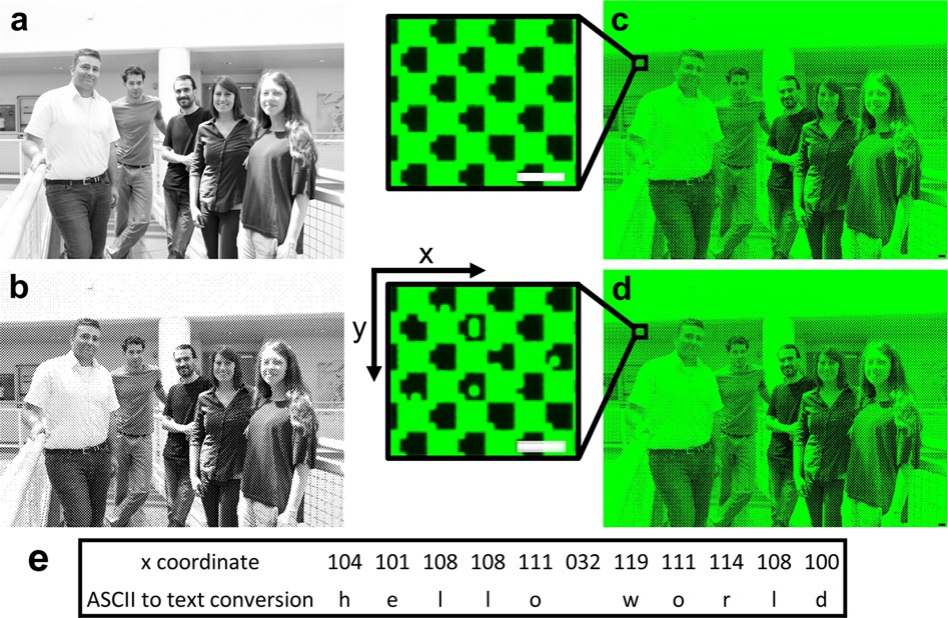
Steganography within a microarray‐made group picture (resolution 1024×768 px). Colors were changed to grayscale (a) followed by pixilation and transformation to a 1 bit bitmap picture (b). Differences in scans obtained after hybridization with a fluorescently labelled d‐DNA probe (c) compared with a mixture of both d‐ and l‐DNA probe (d) are not discernible at first glance (scale bars: 300 μm). Close‐ups of the hidden message section are representing ≈0.07 % of the entire synthesis area and reveal the presence of l‐DNA oligonucleotides at selected *x*,*y* coordinates (scale bars: 100 μm). e) The message can be deciphered by conversion of the *x* coordinates to text.

In summary, we presented the addition of l‐DNA phosphoramidites to our toolbox of building blocks available for photolithographic in situ synthesis of microarrays. We show that the biophysical properties of mirror‐image DNA, including homochiral hybridization behavior and increased nuclease stability remain valid for microarray‐synthesized oligonucleotides. The fluorescently labelled probes required for on‐array hybridization are generated on a separate microarray, cleaved and retrieved in solution, which opens the way to the preparation of large l‐DNA libraries. We then explore a new avenue for l‐DNA as a bioorthogonal hybridization tool in the creation of two‐dimensional binary patterns containing authentication and encrypted messages. Chimeric l‐/d‐DNA microarrays can thus form two independent information channels that can each be accessed separately by hybridization to fluorescently labelled probes. Within standard d‐DNA oligonucleotide arrays, l‐DNA features were designed to form QR codes on the array that may reveal synthesis data as well as provide decoding keys for encrypted information stored on d‐DNA. Forgery‐proof l‐DNA watermarks can be used to confirm authenticity, and sensitive data can be concealed as code in the coordinates of complex synthetic array patterns. The use of mirror‐image oligonucleotides in these applications as add‐ons to common microarrays does not only offer an additional level of pure synthetic complexity, but the clear bioorthogonality between l‐ and d‐enantiomers also brings the prospect for parallelized assays to be performed on surface‐bound l‐/d‐oligo libraries, such as in DNA‐based logic circuits.

## Conflict of interest

The authors declare no conflict of interest.

## Supporting information

As a service to our authors and readers, this journal provides supporting information supplied by the authors. Such materials are peer reviewed and may be re‐organized for online delivery, but are not copy‐edited or typeset. Technical support issues arising from supporting information (other than missing files) should be addressed to the authors.

Supplementary

## References

[1] M. Schena , D. Shalon , R. W. Davis , P. O. Brown , Science 1995, 270, 467–470. 7569999 10.1126/science.270.5235.467

[2] P. L. Ståhl , F. Salmen , S. Vickovic , A. Lundmark , J. F. Navarro , J. Magnusson , S. Giacomello , M. Asp , J. O. Westholm , M. Huss , A. Mollbrink , S. Linnarsson , S. Codeluppi , A. Borg , F. Ponten , P. I. Costea , P. Sahlen , J. Mulder , O. Bergmann , J. Lundeberg , J. Frisen , Science 2016, 353, 78–82. 27365449 10.1126/science.aaf2403

[3] G. Pardatscher , M. Schwarz-Schilling , S. S. Daube , R. H. Bar-Ziv , F. C. Simmel , Angew. Chem. Int. Ed. 2018, 57, 4783–4786; 10.1002/anie.201800281 29469991

[4a] E. M. LeProust , B. J. Peck , K. Spirin , H. B. McCuen , B. Moore , E. Namsaraev , M. H. Caruthers , Nucleic Acids Res. 2010, 38, 2522–2540; 20308161 10.1093/nar/gkq163 PMC2860131

[4b] N. Svensen , J. J. Diaz-Mochon , M. Bradley , PLoS ONE 2011, 6, e24906; 21966380 10.1371/journal.pone.0024906 PMC3179494

[4c] T. L. Schmidt , B. J. Beliveau , Y. O. Uca , M. Theilmann , F. Da Cruz , C.-T. Wu , W. M. Shih , Nat. Commun. 2015, 6, 8634. 26567534 10.1038/ncomms9634 PMC4660042

[5] S. M. Chirieleison , P. B. Allen , Z. B. Simpson , A. D. Ellington , X. Chen , Nat. Chem. 2013, 5, 1000–1005. 24256862 10.1038/nchem.1764 PMC3970425

[6a] N. Goldman , P. Bertone , S. Chen , C. Dessimoz , E. M. LeProust , B. Sipos , E. Birney , Nature 2013, 494, 77–80; 23354052 10.1038/nature11875 PMC3672958

[6b] R. N. Grass , R. Heckel , M. Puddu , D. Paunescu , W. J. Stark , Angew. Chem. Int. Ed. 2015, 54, 2552–2555; 10.1002/anie.201411378 25650567

[7] K. Hölz , E. Schaudy , J. Lietard , M. M. Somoza , Nat. Commun. 2019, 10, 3805. 31444344 10.1038/s41467-019-11670-3 PMC6707258

[8] S. Singh-Gasson , R. D. Green , Y. Yue , C. Nelson , F. Blattner , M. R. Sussman , F. Cerrina , Nat. Biotechnol. 1999, 17, 974–978. 10504697 10.1038/13664

[9] J. Lietard , H. Abou Assi , I. Gomez-Pinto , C. Gonzalez , M. M. Somoza , M. J. Damha , Nucleic Acids Res. 2017, 45, 1619–1632. 28100695 10.1093/nar/gkw1357 PMC5389548

[10a] J. Lietard , D. Ameur , M. J. Damha , M. M. Somoza , Angew. Chem. Int. Ed. 2018, 57, 15257–15261; 10.1002/anie.201806895 PMC6237118 30187993

[10b] C.-H. Wu , M. T. Holden , L. M. Smith , Angew. Chem. Int. Ed. 2014, 53, 13514–13517; 10.1002/anie.201408747 PMC4319565 25339581

[11] P. L. T. Tran , R. Moriyama , A. Maruyama , B. Rayner , J.-L. Mergny , Chem. Commun. 2011, 47, 5437–5439. 10.1039/c1cc11293g 21483923

[12] H. Urata , K. Shinohara , E. Ogura , Y. Ueda , M. Akagi , J. Am. Chem. Soc. 1991, 113, 8174–8175.

[13] S. Fujimori , K. Shudo , Y. Hashimoto , J. Am. Chem. Soc. 1990, 112, 7436–7438.

[14a] A. Garbesi , M. L. Capobinanco , F. P. Colonna , L. Tondelli , F. Arcamone , G. Manzini , C. W. Hilbers , J. M. E. Aelen , M. J. J. Blommers , Nucleic Acids Res. 1993, 21, 4159–4165; 8414968 10.1093/nar/21.18.4159 PMC310044

[14b] K. Hoehlig , L. Bethge , S. Klussmann , PLoS ONE 2015, 10, e0115328. 25679211 10.1371/journal.pone.0115328 PMC4334536

[15a] F. Morvan , C. Génu , B. Rayner , G. Gosselin , J.-L. Imbach , Biochem. Biophys. Res. Commun. 1990, 172, 537–543; 2173569 10.1016/0006-291x(90)90706-s

[15b] Y. Hashimoto , N. Iwanami , S. Fujimori , K. Shudo , J. Am. Chem. Soc. 1993, 115, 9883–9887;

[15c] N. C. Hauser , R. Martinez , A. Jacob , S. Rupp , J. D. Hoheisel , S. Matysiak , Nucleic Acids Res. 2006, 34, 5101–5111. 16990248 10.1093/nar/gkl671 PMC1636439

[16a] M. J. Damha , P. A. Giannaris , P. Marfey , Biochemistry 1994, 33, 7877–7885; 8011650 10.1021/bi00191a015

[16b] W. Zhong , J. T. Sczepanski , ACS Sens. 2019, 4, 566–570; 30843691 10.1021/acssensors.9b00252

[16c] L. Cui , R. Peng , T. Fu , X. Zhang , C. Wu , H. Chen , H. Liang , C. J. Yang , W. Tan , Anal. Chem. 2016, 88, 1850–1855; 26691677 10.1021/acs.analchem.5b04170 PMC4892185

[16d] K. P. Williams , X. H. Liu , T. N. Schumacher , H. Y. Lin , D. A. Ausiello , P. S. Kim , D. P. Bartel , Proc. Natl. Acad. Sci. USA 1997, 94, 11285–11290. 9326601 10.1073/pnas.94.21.11285 PMC23443

[17] N. M. Adams , W. E. Gabella , A. N. Hardcastle , F. R. Haselton , Anal. Chem. 2017, 89, 728–735. 28105843 10.1021/acs.analchem.6b03291 PMC6431534

[18] K.-R. Kim , T. Lee , B.-S. Kim , D.-R. Ahn , Chem. Sci. 2014, 5, 1533–1537.

[19] C. Dose , D. Ho , H. E. Gaub , P. B. Dervan , C. H. Albrecht , Angew. Chem. Int. Ed. 2007, 46, 8384–8387; 10.1002/anie.200703007 17899583

[20] B. E. Young , J. T. Sczepanski , ACS Synth. Biol. 2019, 8, 2756–2759. 31670930 10.1021/acssynbio.9b00335 PMC6953401

[21] J. An , J. Choi , D. Hwang , J. Park , C. W. Pemble , T. H. M. Duong , K.-R. Kim , H. Ahn , H. S. Chung , D.-R. Ahn , Chem. Commun. 2020, 56, 2186–2189. 10.1039/c9cc09351f 31971182

[22a] M. Wang , W. Jiang , X. Liu , J. Wang , B. Zhang , C. Fan , L. Liu , G. Pena-Alcantara , J.-J. Ling , J. Chen , T. F. Zhu , Chem 2019, 5, 848–857;

[22b] J. Weidmann , M. Schnolzer , P. E. Dawson , J. D. Hoheisel , Cell Chem. Biol. 2019, 26, 645–651; 30880154 10.1016/j.chembiol.2019.02.008

[22c] A. Pech , J. Achenbach , M. Jahnz , S. Schulzchen , F. Jarosch , F. Bordusa , S. Klussmann , Nucleic Acids Res. 2017, 45, 3997–4005. 28158820 10.1093/nar/gkx079 PMC5605242

[23] M. Sack , K. Hölz , A.-K. Holik , N. Kretschy , V. Somoza , K.-P. Stengele , M. M. Somoza , J. Nanobiotechnology 2016, 14, 14. 26936369 10.1186/s12951-016-0166-0 PMC4776362

[24] N. Kretschy , A.-K. Holik , V. Somoza , K.-P. Stengele , M. M. Somoza , Angew. Chem. Int. Ed. 2015, 54, 8555–8559; 10.1002/anie.201502125 PMC4531821 26036777

[25] G. H. McGall , A. D. Barone , M. Diggelmann , S. P. A. Fodor , E. Gentalen , N. Ngo , J. Am. Chem. Soc. 1997, 119, 5081–5090.

[26] M. T. Holden , L. M. Smith , ACS Comb. Sci. 2019, 21, 562–567. 31276622 10.1021/acscombsci.9b00088 PMC6701472

