# Supplementary material for: l‐DNA Duplex Formation as a Bioorthogonal Information Channel in Nucleic Acid‐Based Surface Patterning
Source: Chemistry. 2020 Oct 14;26(63):14310–4. doi: 10.1002/chem.202001871 (PMC7702103; doi:10.1002/chem.202001871)
Supplement: Supplementary file 1 — Supplementary [file CHEM-26-14310-s001.pdf]

Chemistry–A European Journal

Supporting Information

L-DNA Duplex Formation as a Bioorthogonal Information Channel in Nucleic Acid-Based Surface Patterning

Erika Schaudy,[a] Mark M. Somoza,*[a, b, c] and Jory Lietard*[a]

SUPPORTING INFORMATION

Table of Contents

General Procedures ... **2**
Microarray functionalization ... 2
Microarray synthesis ... 2
Synthesis of the complementary labelled probes ... 3
Hybridization ... 3
Endonuclease degradation ... 4
Software used ... 4
Investigation of Synthesis Parameters for L-DNA Synthesis ... **4**
Coupling time ... 4
Coupling efficiency ... 4
Photolysis efficiency ... 5
QR Code ... **6**
Authenticity Watermark ... **6**
Concealment of Information in Complex Two-Dimensional Patterns ... **7**
Generation of digital masks for synthesis ... 7
Hybridization and data analysis ... 7
References ... **7**
Author Contributions ... **8**

General Procedures

Microarray functionalization

Microarray synthesis was performed on functionalized Schott NEXTERION glass D slides. Briefly, glass slides were functionalized with 2% *N*-(3-triethoxysilylpropyl)-4-hydroxybutyramide (95%; abcr) in a 95:5 solution of ethanol/water with 0.1% acetic acid for 4 h, followed by two washing steps with an ethanol/water/acetic acid mix (95:5:0.1) and cured at 120 °C under vacuum for 2 h. The method described here produces two arrays with mirror-image sequence layout, but otherwise identical microarrays in parallel[1]. In order to allow for reagents and solvents to flow between the two surfaces, one glass slide must be drilled with one entry and one exit hole followed by washing in an ultrasonic bath for 30 min prior to functionalization.

Microarray synthesis

The synthesis system consists of an Expedite 8909 nucleic acid synthesizer to pump reagents and solvents – anhydrous acetonitrile, activator (0.25 M 4,5-dicyanoimidazole in acetonitrile), oxidizer (tetrahydrofuran/water/pyridine/iodine 90.54/9.05/0.41/0.43 (v/v/v/w); Sigma-Aldrich), exposure solvent (1% imidazole in DMSO; all organic solvents from Biosolve), cyanoethyl phosphoramidites and helium for drying – to the reaction chamber. The second part consists of an optical system, in which UV light produced from a 365 nm high-power UV-LED source (Nichia NVSU333A)[2] is focused on a digital micromirror device (Texas Instruments 0.7 XGA DMD) with 1024 × 768 individually addressable micromirrors. The micromirrors can adopt two positions, one reflecting the incoming UV light onto the synthesis surface *via* an Offner relay, the other tilted position reflects UV light away from the synthesis area. The actual layout of the synthesis area, and therefore the number and location of mirrors addressed in light-directed oligonucleotide synthesis, varied depending on the experiment but the design of the layout was produced using MATLAB (R2011b) and a program made in-house.

Cyanoethyl phosphoramidites were obtained from Orgentis (5′-BzNPPOC D-DNA phosphoramidites), ChemGenes (5′-NPPOC L-DNA and 5′-NPPOC D-DNA phosphoramidites, cleavable dT) and LINK (DMTr-dT), respectively, and used as 0.03 M solutions in dry acetonitrile. Standard coupling times of 15 s for D-DNA and 60 s for mirror-image phosphoramidites were applied. Cyanine-3-CE phosphoramidite (LINK) used for terminal labelling was prepared at a 0.05 M concentration and coupled twice for 300 s each. For the direct comparison of biophysical properties of L- vs D-DNA, the sequence 5′-GTCATCATCATGAACCACCCTGGTCTTTTT-3′ was synthesized in both chemistries on a single microarray. T_5 on the 3′-end serves as linker spacing the oligo of interest from the glass surface, whereas the remaining 25mer represents our quality control sequence for hybridization, designated "QC25". D- and L-DNA features were distributed randomly across the synthesis area to simplify retrieval of fluorescence intensity data. In order to allow for several experiments on a single slide, a layout comprising of four individually addressable, identical subarrays was used in synthesis. In addition, a more illustrative layout was used to illustrate differences in chirality, specificity of hybridization and endonuclease degradation: A photograph of two hands served as mask for synthesis of the L- (left hand) and D-DNA (right hand) 25mer as well as the superimposition of both (3′-L_{25}-D_{25}-5′). Cyanoethyl and base protecting groups were removed by treating the microarrays for 2 h in a 1:1 solution of ethylenediamine/ethanol.

Synthesis of the complementary labelled probes

Fluorescently labelled DNA probes required in hybridization assays were synthesized as regular microarrays on top of a single base-cleavable NPPOC-dT phosphoramidite (T^{cleav}), which was coupled immediately following synthesis of the linker[3]. A final coupling with Cy3 phosphoramidite at the 5′ end served as the labelling method. For instance, the sequence 5′-Cy3-GACCAGGGTGGTTCATGATGATGACT^{cleav}TTTTT-3′ yields a Cy3-labelled probe complementary to the on-array QC25 sequence ("QCcomp-Cy3") after cleavage of the T^{cleav} unit. The entire synthesis area was used to prepare a single probe. Light exposure was such as to reach 99% photodeprotection efficiency and thus increase sequence fidelity. Cleavage of the oligonucleotide from the glass surface was performed by applying 100 µL concentrated NH_4OH (Sigma-Aldrich) directly on the synthesis area and collecting the solution (see Figure S1). This procedure was repeated twice. In a final step, 100 µL NH_4OH were applied for 10 min before collecting the solution again. The combined 400 µL were incubated at 55 °C for 2.5 h to deprotect the nucleobases. After evaporation of the residual NH_3 at 60 °C, the solution was dried down under vacuum. Quantification as well as determination of the Cy3 labelling efficiency was performed using a NanoDrop One (Thermo Scientific) spectrophotometer. Pierce C18 tips (Thermo Scientific) were then used for a quick purification according to an established protocol[4]. Briefly, the probe solution was buffered with triethylammonium acetate (TEAA) to 0.1 M. The C18 resin was first wetted with H_2O/ACN 1:1, then equilibrated with 0.1 M TEAA before loading the sample, followed by washing steps with 0.1 M TEAA and water. Finally, the oligo was eluted in H_2O/ACN 1:1. After drying and re-quantification, the probes were directly used for hybridization to the complementary on-array DNA.

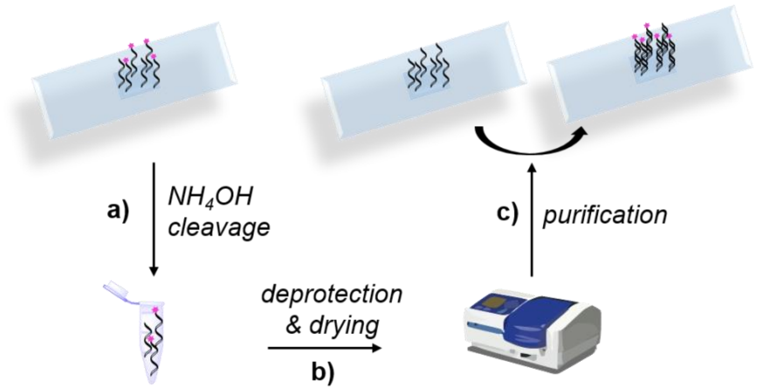

Figure S1. Workflow for the generation of probes used in hybridization experiments. The key steps are the cleavage and collection of a labelled DNA synthesized *in situ* on a microarray (a), removal of protecting groups and quantification (b) and use of the quickly purified and quantified probe for hybridization to complementary DNA on another microarray (c).

Hybridization

Hybridization with Cy3-labelled probes was performed in MES 1× buffer (100 mM MES, 1 M Na^+, 20 mM EDTA, 0.01% Tween-20) supplemented with acetylated BSA (0.44 mg/mL) in custom-made self-adhesive chambers (Grace Biolabs), adapted for the particular layout used in synthesis, for 1 h at 42 °C with rotation. The similarity in solubility and stability allowed for parallel use of L- and D-oligonucleotides of the same sequence in a one-pot hybridization reaction on chimeric

L-/D-DNA microarrays under standard hybridization conditions. After hybridization, the microarrays were washed for 2 minutes in non-stringent washing buffer (6× SSPE, 0.01% Tween-20), 1 minute in stringent washing buffer (100 mM MES, 0.1 M Na^+, 0.01% Tween-20) and 10 seconds in final washing buffer (0.1× SSC). After drying in a microarray centrifuge, they were scanned with a GenePix Personal 4100A scanner (Axon Instruments) at 5 µm resolution and a wavelength of 532 nm.

Probes used in hybridization experiments were generated as described above, except for the group picture array (Figure 4), for which a pure Cy3-labelled D-DNA oligonucleotide was used (Eurogentec). Since each feature of the 1024 × 768 DMD carried potential information, scanning was performed with a GenePix 4400A scanner (Molecular Devices) at a higher, 2.5 µm resolution.

Endonuclease degradation

The susceptibility of DNA on the array towards endonuclease-mediated degradation was tested with 0.1 U/µL TURBO DNase (Invitrogen) in the supplied buffer. A so far unused subarray was first hybridized to L- and D-complements in a self-adhesive chamber, and the nuclease-buffer mix was then applied to the array, followed by incubation for six hours at 37 °C with rotation. After incubation, stringency washes were performed as for hybridization experiments. The array was scanned, then rinsed with pure water for five minutes at room temperature. Another hybridization was performed with a mix of L- and D-DNA QC25comp-Cy3 oligo (0.1 pmol each). After stringency washes, the array was scanned at 532 nm. Degradation assays were also performed on microarray layouts with motifs (Figure 1e). Figure S2 illustrates L- and D-DNA synthesis with footprints, the loss of only D-DNA upon endonuclease degradation representative of the disappearance of the right foot, whereas the left foot (L-DNA) is still visible after the second hybridization.

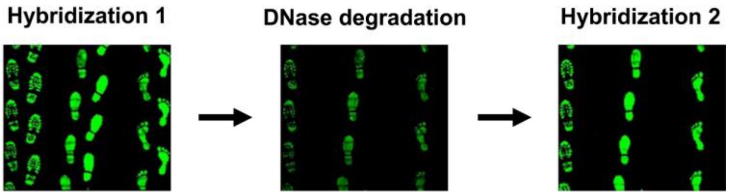

Figure S2. Effect of TURBO DNase degradation. Scan of the pattern of left and right footprints after hybridization with a mix of fluorescently labelled L- and D-DNA (left), directly after enzymatic degradation (center) and following repetition of the initial hybridization (right).

Software used

Adobe Photoshop CS5 was used to generate custom-made patterned synthesis masks to be integrated as templates in MATLAB (R2011b) to produce the corresponding synthesis design files. Scans were aligned to the design files using NimbleScan 2.1.68 (NimbleGen Systems Inc.). The extracted data was then analyzed in Excel and SigmaPlot 12.3. Structures and schemes were created using ChemDraw 19.0 and PowerPoint.

Investigation of Synthesis Parameters for L-DNA Synthesis

Coupling time

Coupling times of 15 s and 60 s, respectively, were compared for L-DNA phosphoramidites on the same microarray. Using a layout which allows for the synthesis of four identical and individually addressable subarrays within the footprint of a full-sized array, two versions (with 15 s and 60 s coupling time) of the same QC25 sequence were synthesized on randomly distributed features and in parallel on a T_5 linker. Comparing the signal intensities after hybridization for the two different coupling times showed a drop in signal intensity of 30% with the shorter coupling time, which interestingly, was not observed for the corresponding NPPOC D-phosphoramidites[5].

Coupling efficiency

The stepwise coupling efficiencies of the monomers were determined using a previously reported terminal labelling approach[6], where Cy3 signal intensity, coupled to the 5′ end of all oligonucleotides, informs on the coupling efficiency. Indeed, signal intensity of the terminal Cy3 is expected to decrease as the number of monomer couplings increases and

follows an exponential decay function y = ae^{-bx}, with y as variable for fluorescence intensity, coefficient a for the maximum signal intensity, x the number of couplings, and 1-b providing the stepwise coupling yield. Briefly, 1 up to 12 consecutive monomers were coupled to a linker, each monomer coupling being followed by a capping step with DMTr-dT phosphoramidite (0.03 M). Varying the linker length from 0 – 11 nucleotides allowed for all oligonucleotides to be of the same final length, irrespective of the number of actual monomer couplings, which prevents Cy3 intensity to vary depending on the distance between the dye and the glass surface. In order to avoid Cy3 signal intensity to be affected by the sequence identity, Cy3 was spaced from the monomers of interest by a 10-nt motif (3′ TTTTTAAAAG), the AAAAG motif yielding the highest Cy3 signal intensities.[7] With high background fluorescence intensity due to non-specific binding of Cy3 to the surface of the array, background features capped with DMTr-dT served as controls. The microarray design allowed for direct comparison of two phosphoramidites at a time (L-dX and D-dX).

Photolysis efficiency

Investigation of the light dose required for efficient photodeprotection was performed according to a previously described method[8] by synthesizing the QC25 sequence and applying a gradient of radiant exposure from 0 up to 11 J/cm², followed by a final hybridization with a Cy3-labelled complementary probe of the same chirality (Figure S3). The two gradients of UV exposure (L- and D-synthesis) result in an increase in hybridization as the UV exposure time increases and the corresponding curve adopts a sigmoidal shape. However, the flatter slope in L-DNA synthesis indicates a delayed photoremoval of the NPPOC group on L-DNA phosphoramidites compared to their D-DNA counterparts. The photolysis efficiency of various photolabile protecting groups had been studied in detail in the past.[9] However, these studies focused on the chemical structures of the protecting groups themselves rather than on the structural differences of the nucleotides they are attached to. To the best of our knowledge, the photodeprotection behavior of L-DNA phosphoramidites has not been discussed so far. Differences in electron energy levels between L- and D-RNA duplexes have been previously described.[10] We thus surmise that chirality could indeed affect photodeprotection efficiency, but it remains unclear at this point. We previously stroke a good balance between efficient photodeprotection and minimized unintended exposure of neighboring features by aiming for 95% photodeprotection efficiency. This threshold is reached for D-DNA synthesis at a radiant exposure of 6 J/cm². In contrast, L-DNA synthesis requires a light dose of 8.6 J/cm² to reach the same photodeprotection efficiency.

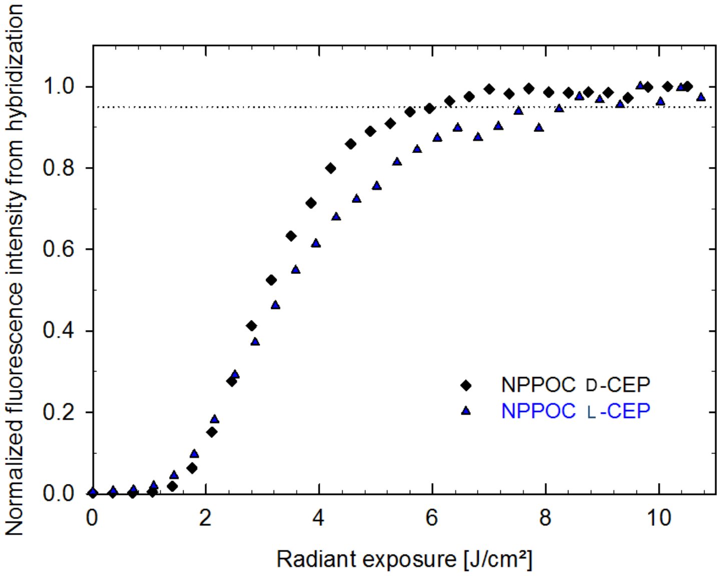

Figure S3. Difference in photolysis efficiency between L- and D-DNA phosphoramidites. Fluorescence intensities after homochiral hybridization to a 25mer with a complementary fluorescently labelled probe were normalized to the highest intensity observed (highest UV light dose). Two arrays with either D- (black diamonds) or L- (blue triangles) monomers were generated with an identical design and a gradient of exposure. Dotted line marks 95% photodeprotection.

QR Code

In order to explore the potential of an L-DNA QR code, the code was manually introduced into the digital masks used in synthesis of four identical subarrays consisting of D-DNA features on a single slide, which is a commonly used layout for microarray synthesis in our lab. A random 128 bit key was generated using an online tool (https://randomkeygen.com/). This key can be used to restrict access to relevant information required for analysis and data extraction from the D-DNA subarrays. The QR code was generated online (https://www.the-qrcode-generator.com/), restricting the size to 50 pixel in order to prevent superimposition with any of the subarrays.
In a proof of principle, only a single D-DNA sequence (QC25) was synthesized in the four subarrays. In order to point out homochiral hybridization as well as differences in stability towards endonucleolytic degradation between the two enantiomers, the QR code was made of exactly the same sequence, but in L-DNA form.
Hybridization with the fluorescently labelled D-DNA complement did not reveal the QR code. Following an additional hybridization including the L-DNA version of the same probe in the mixture, the QR code turned visible. Testing QR code scanners on different smartphones showed that the D-DNA grid crossing the code was preventing proper QR scanning for some applications, but successful readout independent of the device used was accomplished after degradation of the D-DNA using TURBO DNase (Figure 2).

Authenticity Watermark

In order to prevent the sequences used for the creation of the watermark to be deciphered and, in so doing, make it forgery-proof, a method for combining oligonucleotide strands of high sequence similarity within the same feature for data encryption was followed[11]. The following three blocks of 10mers were used to generate the sequences:

A: 5′-CGGACGGCTA
B: 5′-GAATATTACC
C: 5′-ATCGAGGACG

The blocks were combined to produce L_x and L_y 30mers and 40mers, respectively, x being either ABC or BCA and y being either ACAC, BCAC or ACAB. These L_x and L_y oligomers were mixed in five different V combinations in order to generate a specific hybridizable pattern within an array of 5×5 features (Table S1). All five versions are designed so that any sequencing by hybridization performed using pentamers would return a signal for hybridization to both L_x and L_y on each feature, thus preventing sequence identity to be exposed. Therefore, the hidden pattern only appears when using the correct fluorescently labelled key probe provided by the manufacturer. L_x and L_y are spaced by a short stretch of D-DNA. In order to prevent SBH to be performed by specifically removing the L_y sequence at the 5′-end in an endonuclease treatment of the D-DNA spacer, a D-DNA T_5 linker was introduced, resulting in the loss of the entire oligonucleotide strand upon degradation.

Table S1. Four different sequences of the blocks A, B and C were combined to generate five versions of the 3′-L_x-D-L_y-5′ chimera (V_1 - V_5) for synthesis of the authenticity watermark. All five combinations are composed of an L-DNA 30mer at the 3′- and a 40mer at the 5′- side, spaced by a D-DNA T_5 linker (_) from one another.

Name	Blocks combined	Scheme L_x-D-L_y	Sequence 5′ -> 3′
V_1	ABC_ ACAC	L_{ABC}-D-L_{ACAC}	ATCGAGGACGCGGACGGCTAATCGAGGACGCGGACGGCTA_ ATCGAGGACGGAATATTACCCGGACGGCTA
V_2	ABC_ BCAC	L_{ABC}-D-L_{BCAC}	ATCGAGGACGCGGACGGCTAATCGAGGACGGAATATTACC_ ATCGAGGACGGAATATTACCCGGACGGCTA
V_3	BCA_ ACAC	L_{BCA}-D-L_{ACAC}	ATCGAGGACGCGGACGGCTAATCGAGGACGCGGACGGCTA_ CGGACGGCTAATCGAGGACGGAATATTACC
V_4	BCA_ ACAB	L_{BCA}-D-L_{ACAB}	GAATATTACCCGGACGGCTAATCGAGGACGCGGACGGCTA_ CGGACGGCTAATCGAGGACGGAATATTACC
V_5	ABC_ ACAB	L_{ABC}-D-L_{ACAB}	GAATATTACCCGGACGGCTAATCGAGGACGCGGACGGCTA_ ATCGAGGACGGAATATTACCCGGACGGCTA

Aiming for a hybridization signal with the Cy3-labelled complementary probe to the ABC block (5'-Cy3-TAGCCGTCCGGGTAATATTCCGTCCTCGAT-3′) as our key, features with V_1, V_2 and V_5 combinations are expected to give a signal. Background features, consisting of just a D-DNA T_5mer, were introduced to create an additional level of intensity in case of cross-hybridization. Distribution of these sequences in a non-randomized fashion allows for creation of, in theory, 6^{25} undecipherable sequence patterns. Authenticity can be validated straightforward upon hybridization, which allows for visualizing a specific pattern that can be identified at first glance. In addition, watermark variation from synthesis batch to batch can be realized easily during the array design process.

Concealment of Information in Complex Two-Dimensional Patterns

We applied here a steganographic approach to patterning nucleic acids on microarrays, by hiding information within an otherwise mundane image prepared at 1024 × 768. Coordinates of an individual set of pixels within the 1024 × 768 pixel matrix were chosen so that their x coordinates, taken by increasing order of their respective y coordinates, would form a string of decimal numbers that can then be converted into text. Those individual pixels would appear upon hybridization to an L-DNA key probe only. The amount of hidden L-DNA features within the D-DNA picture is small enough (11 pixels out of 786,432 pixels in total) so as to remain virtually undetectable to the naked eye when inspecting the D-hybridized and the L-/ D-hybridized versions. Detection of these coding pixels requires not only knowledge of sequence and chemistry of the fluorescently labelled probe, but also access to the original design file and software for alignment.

Generation of digital masks for synthesis

A picture of our working group was modified to fit the dimensions of the digital micromirror device (1024 × 768 pixels), using all individual mirrors available for synthesis. The color scheme of the picture was changed to grayscale, then to a 1 bit bitmap file (threshold 50 %) to generate a black-and-white picture that the DMD can understand as a digital mask. Every white pixel then becomes a synthetic feature for a D-DNA QC25mer. Individual pixels, localized within the black background of the digital mask, were selected and switched to white, where synthesis of the L-DNA QC25mer would be allowed to happen due to the corresponding micromirror being switched to an “ON”-position. A design file for the steganography picture was manually generated, which included the introduction of four anchor points as fiducial features to allow for alignment of the scans.

Hybridization and data analysis

Hybridization after synthesis was first performed using 1.3 pmol of Cy3-labelled QC25comp D-DNA probe, then 0.09 pmol L-DNA probe was added for another round of hybridization. The differences between the high-resolution scans (Figure 4) are imperceptible to the naked eye. Knowing the coordinates of the hidden code allows to zoom in on the area of interest, where at 800 % zoom additional pixels become evident. The exact coordinates of these pixels can be obtained by aligning scans with the original design file. Comparing the readout files of the two hybridization steps and filtering for unusual differences reveal the x and y coordinates of the designated L-DNA pixels. An additional level of complexity is added in the code whereby the receiver needs to perform a single digit subtraction for all coordinates identified. Coordinates given away in Figure 4e have already been corrected for this factor. Feeding the x coordinates ordered by increasing value of the corresponding y coordinate into a decimal-to-text converter results in a short message hidden by us (Table S2), which we were able to successfully retrieve.

Table S2. Code decrypted by detection of an increase in signal intensity in pixels originally labelled as black background spots upon hybridization with an L-DNA complementary probe to a microarray patterned with a picture at 1024 × 768 resolution.

y coordinate	x coordinate	x - 1	Decimal to text conversion (ASCII)
178	105	104	h
179	102	101	e
180	109	108	l
181	109	108	l
183	112	111	o
184	033	032	
185	120	119	w
186	112	111	o
188	115	114	r
189	109	108	l
190	101	100	d

References

[1] M. Sack, N. Kretschy, B. Rohm, V. Somoza, M. M. Somoza, *Anal. Chem.* **2013**, *85*, 8513-8517.
[2] K. Hölz, J. Lietard, M. M. Somoza, *ACS Sustain. Chem. Eng.* **2017**, *5*, 828-834.
[3] J. Lietard, N. Kretschy, M. Sack, A. S. Wahba, M. M. Somoza, M. J. Damha, *Chem. Commun.* **2014**, *50*, 12903-12906.
[4] J. Lietard, E. Schaudy, K. Hölz, D. Ameur, M. M. Somoza, *J. Vis. Exp.* **2019**, e59936.
[5] M. Sack, K. Hölz, A.-K. Holik, N. Kretschy, V. Somoza, K.-P. Stengele, M. M. Somoza, *J. Nanobiotechnology* **2016**, *14*, 14.
[6] a) J. Lietard, D. Ameur, M. J. Damha, M. M. Somoza, *Angew. Chem. Int. Ed.* **2018**, *57*, 15257-15261; b) G. H. McGall, A. D. Barone, M. Diggelmann, S. P. A. Fodor, E. Gentalen, N. Ngo, *J. Am. Chem. Soc.* **1997**, *119*, 5081-5090.

[7] N. Kretschy, M. M. Somoza, *PLoS ONE* **2014**, *9*, e85605.
[8] N. Kretschy, A.-K. Holik, V. Somoza, K.-P. Stengele, M. M. Somoza, *Angew. Chem. Int. Ed.* **2015**, *54*, 8555-8559.
[9] A. Hasan, K.-P. Stengele, H. Giegrich, P. Cornwell, K. R. Isham, R. A. Sachleben, W. Pfleiderer, R. S. Foote, *Tetrahedron* **1997**, *53*, 4247-4264.
[10] S. Bolik, M. Rubhausen, S. Binder, B. Schulz, M. Perbandt, N. Genov, V. Erdmann, S. Klussmann, C. Betzel, *RNA* **2007**, *13*, 1877-1880.
[11] M. T. Holden, L. M. Smith, *ACS Comb. Sci.* **2019**, *21*, 562-567.

Author Contributions

E.S. performed the experiments and analyzed the data. J.L. and M.M.S conceived the experiments. E.S., J.L. and M.M.S. wrote the manuscript.
